# Scaffold Role of DUSP22 in ASK1-MKK7-JNK Signaling Pathway

**DOI:** 10.1371/journal.pone.0164259

**Published:** 2016-10-06

**Authors:** Anna Ju, Young-Chang Cho, Ba Reum Kim, Sung Goo Park, Jeong-Hoon Kim, Kwonseop Kim, Jaehwi Lee, Byoung Chul Park, Sayeon Cho

**Affiliations:** 1 College of Pharmacy, Chung-Ang University, Seoul, 06974, Republic of Korea; 2 Disease Target Structure Research Center, Korea Research Institute of Bioscience and Biotechnology, Daejeon, 34141, Republic of Korea; 3 Personalized Genomic Medicine Research Center, Korea Research Institute of Bioscience and Biotechnology, Daejeon, 34141, Republic of Korea; 4 College of Pharmacy and Research Institute for Drug Development, Chonnam National University, Gwangju, 61186, Republic of Korea; University of Leeds Faculty of Medicine and Health, UNITED KINGDOM

## Abstract

Mitogen-activated protein kinases (MAPKs) are involved in a variety of intracellular events such as gene expression, cell proliferation, and programmed cell death. MAPKs are activated by dual phosphorylation on threonine and tyrosine residues through sequential activation of protein kinases. Recent studies have shown that the protein kinases involved in MAPK signal transductions might be organized into signaling complexes by scaffold proteins. These scaffold proteins are essential regulators that function by assembling the relevant molecular components in mammalian cells. In this study, we report that dual-specificity phosphatase 22 (DUSP22), a member of the protein tyrosine phosphatase family, acts as a distinct scaffold protein in c-Jun N-terminal kinase (JNK) signaling. DUSP22 increased the phosphorylation in the activation loop of JNK regardless of its phosphatase activity but had no effect on phosphorylation levels of ERK and p38 in mammalian cells. Furthermore, DUSP22 selectively associated with apoptosis signal-regulating kinase 1 (ASK1), MAPK kinase 7 (MKK7), and JNK1/2. Both JNK phosphorylation and JNK-mediated apoptosis increased in a concentration-dependent manner regardless of DUSP22 phosphatase activity at low DUSP22 concentrations, but then decreased at higher DUSP22 concentrations, which is the prominent feature of a scaffold protein. Thus, our data suggest that DUSP22 regulates cell death by acting as a scaffold protein for the ASK1-MKK7-JNK signal transduction pathway independently of its phosphatase activity.

## Introduction

Mitogen-activated protein kinases (MAPKs) regulate a vast array of physiological processes such as gene expression, cell proliferation, and programmed cell death in response to extracellular stimuli including growth factors, nutrient status, stress, or inductive signals [1–3]. In mammalian cells, three major groups of MAPKs have been characterized—extracellular signal-related kinases (ERKs), c-Jun N-terminal kinases (JNKs), p38 MAPKs. MAPK modules are composed of three distinct kinases called MAPK, MAPK kinase (MAP2K), and MAPK kinase kinase (MAP3K) [4]. MAPKs are activated by dual phosphorylation on threonine and tyrosine residues through signaling cascades; this phosphorylation induces conformational changes in MAPKs, which leads to enhancement of their catalytic activity [5]. Protein phosphatases, which are classified into several groups according to their substrate specificity, dephosphorylate the phospho-tyrosine and/or phospho-serine/threonine of their substrates, which indicates that protein phosphatases are critical in regulating the magnitude and duration of MAPK activity [6–8]. MAPK signaling modules can be organized into signaling complexes by scaffold proteins. These scaffold proteins determine the localization of the signal components to specific cellular sites or substrates and provide spatial organization for the regulation of cascade activation [9]. A number of scaffold proteins that contribute to the regulation of MAPK pathways have been discovered; e.g., JIP1, JSAP1, MP1, KSR, and β-arrestin 2 [10–14].

The dual-specificity phosphatase (DUSP) family, a subset of protein tyrosine phosphatases (PTPs), is classified into two subgroups according to the presence of a kinase-interacting motif [15]. DUSP22, a member of the low molecular weight atypical DUSP group that lacks a kinase-interacting motif, is ubiquitously expressed in mammalian cells [16]. DUSP22 negatively regulates the estrogen receptor-α-mediated signaling pathway and interleukin 6 (IL-6)-leukemia inhibitory factor (LIF)-signal transducer and activator of transcription 3 (STAT3)-mediated signaling pathway [17, 18]. Recently, Yu et al. showed that DUSP22 expression is correlated with tumor size in colorectal cancer [19].

DUSP22 has been also reported to regulate MAPK signal transduction. The effect of DUSP22 on MAPKs, however, is controversial since there have been several conflicting reports regarding its substrate specificity. One report showed that DUSP22 dephosphorylates ERK2 *in vitro* [20] while other studies showed that DUSP22 enhances JNK activation but not p38 and ERK2 [21, 22]. Little is known about the functional roles of DUPS22 and the underlying mechanisms. Therefore, further studies are required to clarify the physiological role of DUSP22.

In this study, we show that DUSP22 regulates JNK activation by acting as a scaffold protein in the modulation of JNK signaling through the formation of the ASK1-MKK7-JNK1/2 complex.

## Materials and Methods

### Cell culture and Transfection

Human embryonic kidney (HEK) 293 and HCT 116 cells were obtained from American Tissue Culture Collection (ATCC, Manassas, VA) and maintained at 37°C in Dulbecco’s modified Eagle’s medium (DMEM, Thermo Scientific, Waltham, MA) supplemented with 10% fetal bovine serum (FBS, Thermo Scientific) and penicillin/streptomycin (Life Technologies Corporation, Carlsbad, CA) in the presence of 5% CO_2_. For transient transfection, 4×10^5^ cells were plated in 60 mm cell culture dish, grown overnight, and transfected with DNA using polyethylenimine (PEI, Polysciences, Inc., Warrington, PA).

### Plasmid constructions

FLAG-DUSP22 WT, HA-DUSP22 WT, FLAG-DUSP22 C88S, FLAG-ASK1, HA-MKK4, HA-MKK7, HA-ERK1, HA-JNK1, HA-JNK2, and HA-p38γ expression plasmids were constructed in pcDNA3.1/Zeo plasmid (Invitrogen, Carlsbad, CA). GST-MKK7 expression plasmid was constructed in pEBG plasmid (Addgene, Cambridge, MA). GST-c-Jun (1–135) expression plasmid was constructed in the pGEX-6P-1 plasmid (Amersham Biosciences, Little Chalfont, UK). HA-ASK1 expression plasmid was a generous gift from Dr. Hidenori Ichijo.

### Reagents and antibodies

Anti-ERK, anti-JNK, anti-p38α/β (A-12), and anti-HA-probe (Y-11) antibodies were purchased from Santa Cruz Biotechnology (Santa Cruz, CA). Anti-phospho-p44/42 MAPK (ERK1/2) (Thr-202/Tyr-204), anti-phospho-SAPK/JNK (specific for phospho-Thr-183 and phospho-Tyr-185), anti-phospho-p38 MAPK (Thr-180/Tyr-182), anti-cleaved PARP (Asp-214), anti-caspase 3, and anti-phospho-c-Jun (Ser-63) antibodies were from Cell Signaling Technology (Danvers, MA). Monoclonal anti-FLAG antibody, anti-FLAG M2 affinity gel, and sodium orthovanadate (Na_3_VO_4_) were from Sigma-Aldrich (St. Louis, MO). Glutathione Agarose 4B was purchased from Incospharm (Daejeon, Korea).

### Co-immunoprecipitation and immunoblot analysis

For binding assays, HEK 293 cells were co-transfected with 1 μg of FLAG-DUSP22 together with 1 μg of HA-MAPKs or HA-MAPKKs expression plasmids. After 48 h, cells were washed twice with phosphate-buffered saline (PBS) and lysed in PTP lysis buffer (20 mM Tris-HCl (pH 7.5), 150 mM NaCl, 1 mM EDTA, 0.5% Triton X-100, 0.5% IGEPAL, 10% glycerol, 1 mM DTT, 1 mM PMSF). Soluble cell lysates from centrifugation were immunoprecipitated with the anti-FLAG M2 affinity gel for 16 h at 4°C using a rotation device. Immunoprecipitated proteins were eluted with the SDS-PAGE sample buffer. Samples were boiled at 100°C for 5 min and run in SDS-10% or 12% polyacrylamide gels and transferred onto nitrocellulose membrane as described [23]. The membrane was blocked in 5% nonfat dry milk and incubated with appropriate antibody with 5% BSA or 5% nonfat dry milk, followed by incubation with a secondary antibody conjugated to horseradish peroxidase. The immunoreactive bands were visualized using an ECL system (Pierce, Rockford, IL).

For the detection of apoptosis by immunoblot analyses, HCT 116 cells at 60% confluence in 60 mm dishes were transfected with various amounts of DUSP22 expression plasmids for 48 h at 37°C. After incubation, total cell lysates were prepared in PTP lysis buffer, mixed with 5 X sample buffer, and then boiled at 100°C for 5 min. The protein samples were subjected to SDS-PAGE and subsequently transferred onto nitrocellulose membrane. The membranes were then subjected to immunoblot analyses.

### *In vitro* phosphatase assay

*In vitro* phosphatase assays were performed as described by Tierno *et al*. [24]. Cells were transfected with 1.5 μg of FLAG-DUSP22 WT or FLAG-DUSP22 C88S expression plasmid. Cleared cell lysates were mixed with the anti-FLAG M2 affinity gel and incubated for 1 h at 4°C using a rotation device. After incubation, immunoprecipitated cell lysates were washed three times with PTP lysis buffer and phosphatase activity was measured using 3-O-methylfluorescein phosphate (OMFP; Sigma-Aldrich) as a substrate. The final incubation mixture (150 μl) was optimized for enzyme activity and composed of 30 mM Tris-HCl (pH 7.0), 75 mM NaCl, 1 mM EDTA, 0.33% BSA with DUSP22 WT or DUSP22 C88S protein. Reactions were initiated by addition of OMFP and incubated for 30 min at 37°C. Fluorescence emission from products was measured at 485 nm (excitation) and 535 nm (emission) using Synergy H1 Microplate Reader (BioTek Instruments, Winooski, VT).

### *In vitro* Kinase assay

HEK 293 cells were co-transfected with 1 μg of FLAG-DUSP22 together with 1 μg of HA-JNK1 expression plasmids. After 48 h, cells were treated with 1 mM H_2_O_2_ for 1 h and then lysed in lysis buffer. Soluble cell lysates from centrifugation were immunoprecipitated with rabbit anti-HA antibody followed by incubation with protein A/G agarose beads for 16 h at 4°C. The beads were washed once with lysis buffer, twice with a buffer (20 mM Tris-HCl (pH 7.5), 150 mM NaCl, 5 mM EDTA, 2 mM DTT, and 1 mM PMSF), and then once with a buffer (20 mM Tris-HCl (pH 7.5), and 20 mM MgCl_2_). The beads were then added with 1 μg of GST-c-Jun (1–135) in 100 μl of kinase reaction buffer (20 mM Tris-HCl (pH 7.5), 20 mM MgCl_2_, and 20 μM ATP), and reaction mixture were incubated at 30°C for 15 min. The reaction was stopped by adding SDS sample buffer and the products of kinase reactions were analyzed by immunoblotting with an anti-phospho-c-Jun (Ser-63) antibody.

### Cell viability assay

HEK 293 cells at 30% confluence in 12-well plates were transfected with 1.0 μg of DUSP22 expression plasmid for 24 h at 37°C. After incubation, cell viability was determined using the EZ-cytox cell viability kit (DAEIL Lab, Seoul, Korea) according to the manufacturer’s protocol. Ez-cytox solution was added to each well. After incubation for 1 h, supernatants were transferred to new 96-well plates and the absorbance at 450 nm (viability value) and 650 nm (reference value) was measured using Synergy H1 Microplate Reader. Cell survival was expressed as the percentage of absorbance (A_450_-A_650_) of the treated cells relative to that of the untreated cells.

### Statistical Analysis

All data were obtained from three independent experiments. *P*-values were calculated by Student’s *t* test and expressed as means ± standard error of the mean (SEM). For multiple comparisons, statistical analysis was performed using the one-way analysis of variance (ANOVA). *P* values < 0.01 were considered to indicate statistical significance.

## Results

### DUSP22 selectively upregulates JNK phosphorylation levels

**Fig 1:**
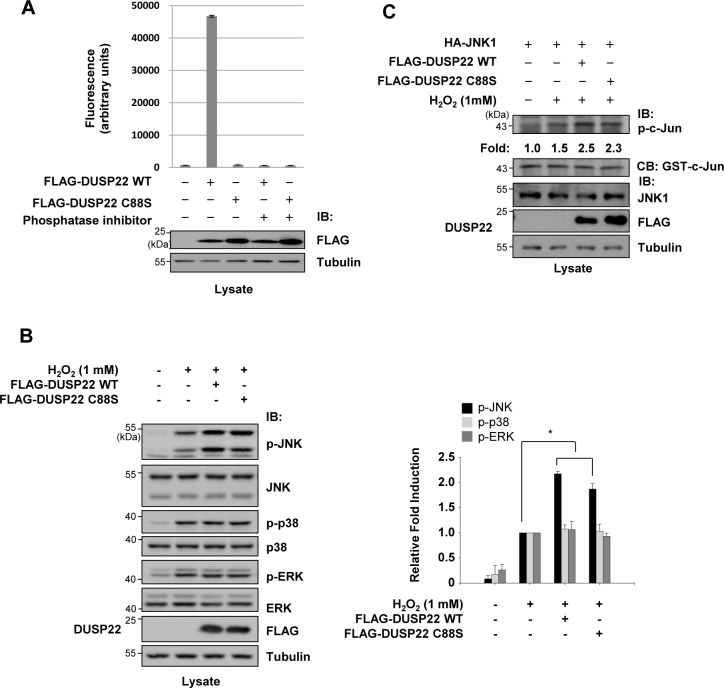
Involvement of DUSP22 in MAPK phosphorylation. (A) HCT 116 cells were transfected with FLAG-DUSP22 WT or FLAG-DUSP22 C88S expression plasmid. After 48 h, cells were lysed in PTP lysis buffer. Cell lysates were immunoprecipitated with the anti-FLAG M2 affinity gel for 1 h at 4°C. Then, phosphatase activity was measured as described in the Materials and methods and presented as fluorescence intensity (in arbitrary unit). Immunoblot analysis was performed with cell lysates using an anti-FLAG or an anti-tubulin antibody. IB, immunoblot. (B) Transfected HCT 116 cells were further incubated in the presence or absence of H_2_O_2_ (1 mM) for 1 h and lysed in PTP lysis buffer. Cell lysates were subjected to immunoblotting with antibodies as indicated. Twenty micrograms of cell lysates were loaded in each lane for detection. Those levels were quantified by analysis with LabWorks software (UVP Inc., Upland, CA) and normalized to corresponding total protein levels. Right panel: Relative fold induction of phosphorylated MAPK level after normalization. Tubulin served as a loading control. All data are representative of three independent experiments. Statistical significance was determined by one-way ANOVA. *P< 0.01 compared with control. (C) HEK 293 cells were co-transfected using HA-JNK1 expression plasmid together with FLAG-DUSP22 WT or FLAG-DUSP22 C88S expression plasmid. After 48 h, cells were additionally incubated in the presence or absence of H_2_O_2_ (1 mM) for 1 h and lysed in lysis buffer. HA-JNK1 was immunoprecipitated with an anti-HA antibody. Immunoprecipitates were subjected to an *in vitro* kinase assay using 1 μg of GST-c-Jun as a substrate. Kinase activity was normalized to the expression level of c-Jun and presented as fold increase. CB, coomassie blue staining.

DUSP22 appears to play a role in the regulation of MAPK signaling; however, the mechanism by which it regulates this pathway is controversial. Although initial studies reported controversial results regarding the substrates of DUSP22 for dephosphorylation, recent reports proposed that DUSP22 activates JNK signaling [15]. However, it is unclear how DUSP22 regulates JNK signal transduction pathways at the molecular level. To determine its molecular mechanism of action in the regulation of MAPK signaling, we investigated whether DUSP22 specifically regulates the phosphorylation levels in the phosphorylation loops of MAPKs in HCT 116 cells. A DUSP22 C88S mutant in which the catalytically critical Cys-88 was replaced by Ser was constructed to determine whether DUSP22 phosphatase activity is required for JNK activation. Expression of DUSP22 C88S protein in HCT 116 cells was immunoprecipitated and assayed for enzymatic activity using *in vitro* phosphatase assays to confirm that it lacks phosphatase activity (Fig 1A). While the phosphatase activity of DUSP22 wild-type (WT) was over 40-fold higher than basal levels, the DUSP22 C88S mutant lacked phosphatase activity. Furthermore, a phosphatase inhibitor (Na_3_VO_4_) completely inhibited the phosphatase activity of DUSP22. Following transfection of HCT 116 cells with FLAG-DUSP22 WT or C88S mutant expression plasmids, the endogenous levels of phosphorylated MAPKs were determined by immunoblot analysis using antibodies specific for phospho-amino acids in the phosphorylation loops. The levels of phospho-JNK (p-JNK) were enhanced in the presence of either the DUSP22 WT or C88S mutant, whereas p-ERK and p-p38 levels were unchanged (Fig 1B), suggesting that the effect of DUSP22 on MAPK signal transduction pathways is specific to JNK signaling and that DUSP22 activity towards JNK activation is phosphatase-independent. As shown in Fig 1C, immunoprecipitated JNK from cells transfected with DUSP22 WT or C88S expression plasmids had higher JNK activity towards to c-Jun phosphorylation than JNK from mock-transfected cells. Collectively, these results indicate that DUSP22 selectively upregulates the phosphorylation levels of JNK independent of its phosphatase activity in cells.

### DUSP22 interacts with ASK1, MKK7, and JNK1/2 independent of its phosphatase activity

**Fig 2:**
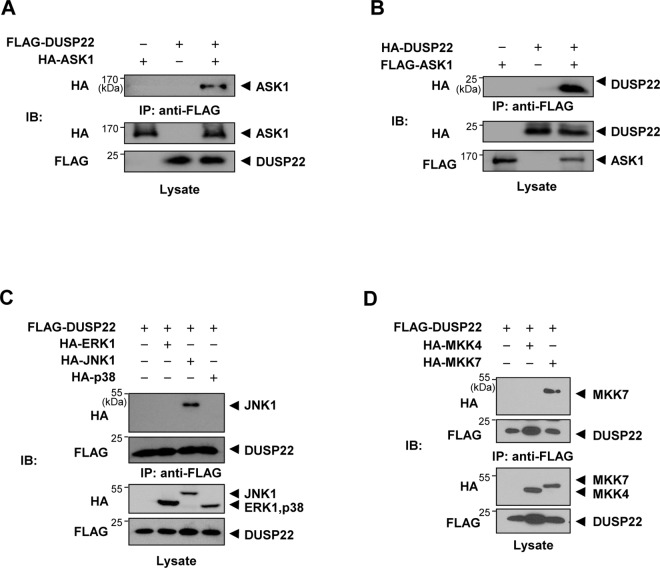
Interaction of DUSP22 with ASK1, JNK1/2, and MKK7. (A) HEK 293 cells were co-transfected using FLAG-DUSP22 expression plasmid with or without HA-ASK1 expression plasmid. After 48 h, lysates were prepared and incubated with the anti-FLAG M2 affinity gel. Pulled-down DUSP22 complexes were subjected to SDS-PAGE. Immunoblot analysis was performed using an anti-HA antibody. IP, immunoprecipitation. (B) HEK 293 cells were co-transfected with HA-DUSP22 expression plasmid with or without FLAG-ASK1 expression plasmid. After immunoprecipitation, pulled-down ASK1 complex was detected as described above. HEK 293 cells were transfected with FLAG-DUSP22 expression plasmid together with (C) HA-ERK1, HA-JNK1, or HA-p38γ (D) HA-MKK4 or HA-MKK7. Cells were lysed in PTP lysis buffer and immunoprecipitated with the anti-FLAG M2 affinity gel. The immunoprecipitates were subjected to SDS-PAGE and then immunoblotting with anti-HA and anti-FLAG antibodies.

Several factors are involved in JNK signal transduction pathways: Apoptosis signal-regulating kinase 1 (ASK1), a member of the MAP3K family, is activated in response to several death stimuli, including TNF, FAS, and reactive oxygen species (ROS) such as hydrogen peroxide. ASK1 regulates both the JNK and p38 pathways by activating MKK4/7 and MKK3/6, respectively [25]. Since DUSP22 plays a positive role in JNK signal transduction, we performed co-immunoprecipitation to investigate whether DUSP22 binds to and regulates JNK signaling molecules. HEK 293 cells were transiently co-transfected with FLAG-DUSP22 and HA-ASK1 expression plasmids. Cell lysates were immunoprecipitated with the anti-FLAG M2 affinity gel, followed by immunoblot analysis with an anti-HA antibody to detect ASK1 in immunoprecipitated DUSP22 complexes (Fig 2A). ASK1 was detected in the immunoprecipitated DUSP22. We also confirmed that FLAG-ASK1 pulled down HA-DUSP22 in a reciprocal manner (Fig 2B). These results show that DUPS22 interacts with ASK1.

Since MKK4 and MKK7, the downstream kinases of ASK1, can activate JNK, we investigated whether DUSP22 could interact with MAPKs including JNK and two JNK activators, MKK4 and MKK7. To confirm the interaction between DUSP22 and MAPKs or MAP2Ks, cells were transiently transfected with FLAG-DUSP22 together with HA-ERK1, HA-JNK1, HA-p38γ, HA-MKK4, or HA-MKK7 plasmids, followed by immunoprecipitation with the anti-FLAG M2 affinity gel and then immunoblotting using an anti-HA antibody. The results showed that DUSP22 selectively interacted with JNK1 among the ERK1, JNK1, and p38γ MAPKs (Fig 2C). In addition, we found that DUSP22 interacted with MKK7, but not with MKK4 (Fig 2D). These data suggest that JNK1 and MKK7 are specific targets of DUSP22.

**Fig 3:**
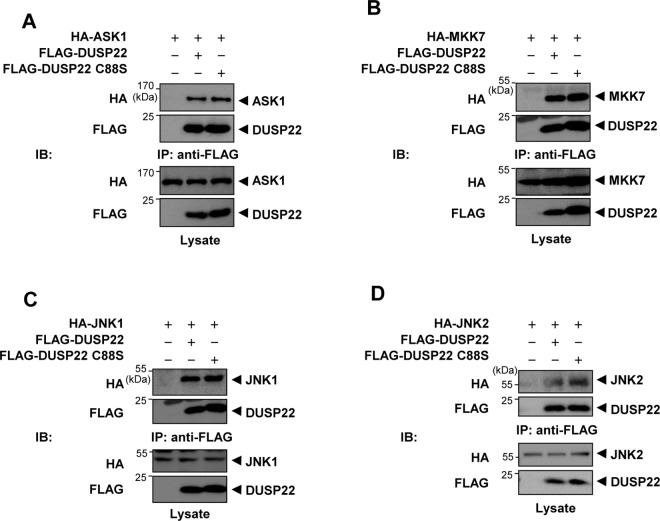
Interaction of DUSP22 mutant with ASK1, MKK7, and JNK1 and dimerization of DUSP22. FLAG-DUSP22 WT or FlAG-DUSP22 C88S expression plasmid was co-transfected together with (A) HA-ASK1, (B) HA-MKK7, (C) HA-JNK1, or (D) HA-JNK2 expression plasmid. After 48 h, cells were lysed in lysis buffer and immunoprecipitated with the anti-FLAG M2 affinity gel. Pulled-down DUSP22 WT and mutant complexes were detected by immunoblotting with anti-HA and anti-FLAG antibodies. After 48 h, cells were lysed and immunoprecipitated with the anti-FLAG M2 affinity gel. The immunoprecipitates were subjected to SDS-PAGE and then immunoblotting with an anti-HA antibody.

Subsequently, to assess whether the phosphatase activity of DUSP22 is crucial for formation of complexes with ASK1, MKK7, or JNK1, respectively, HEK 293 cells were co-transfected with FLAG-DUSP22 WT or FLAG-DUSP22 C88S together with HA-ASK1, HA-MKK7, or HA-JNK1. Cell lysates were immunoprecipitated with the anti-FLAG M2 affinity gel and then immunoblotting using an anti-HA antibody. The DUSP22 mutant also associated with ASK1, MKK7, and JNK1 which are components of the JNK signaling pathway (Fig 3A, 3B, and 3C). Furthermore, JNK2 was also able to associate with DUSP22 WT or C88S (Fig 3D). Taken together, our results suggest that DUSP22 forms a complex with ASK1, MKK7, and JNK1/2, which might facilitate signaling by bringing the signaling molecules together.

### DUSP22 acts as a scaffold protein in ASK1-MKK7-JNK signaling

Scaffold proteins contribute to the specificity of signal transduction cascades by assembling functionally associated signaling partner proteins into specific biochemical pathways [26]. Since we confirmed that DUSP22 interacts with ASK1, MKK7, and JNK and enhances JNK activation, and DUSP22 might act as a platform for recruiting signaling components of a cascade into complexes to regulate signaling strength, we hypothesized that DUSP22 acts as a scaffold protein for the JNK signaling pathway.

**Fig 4:**
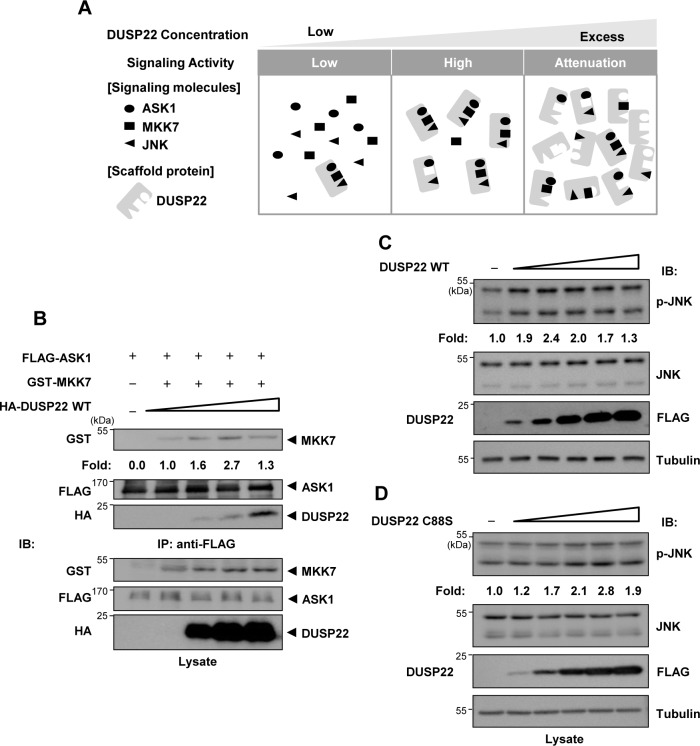
Scaffold role of DUSP22. (A) Tethering mechanism of DUSP22 as a scaffold protein. (B) HEK 293 cells were co-transfected using 3 μg each of FLAG-ASK1 and GST-MKK7 expression plasmids in 100 mm tissue culture dishes and then split into 60 mm tissue-culture dishes. After 24 h, HA-DUSP22 (0, 0.75, 1.5, 3 μg) expression plasmid was transfected in a dose-dependent manner into HEK 293 cells. After 48 h of transfection, cells were lysed and immunoprecipitated with the anti-FLAG M2 affinity gel. Then, the immunoprecipitates were subjected to SDS-PAGE and then immunoblotting with an anti-HA, anti-GST, or anti-FLAG antibody. (C) FLAG-DUSP22 WT (0, 0.5, 0.75, 1.25, 2, 3 μg) or (D) FLAG-DUSP22 C88S (0, 0.4, 0.6, 1, 1.6, 2.4 μg) expression plasmid was transiently transfected in a dose-dependent manner into HEK 293 cells. After 48 h, cell lysates were subjected to immunoblotting with anti-JNK and anti-p-JNK antibodies. Relative JNK phosphorylation levels were quantified by analysis with LabWorks software and normalized to corresponding total JNK protein levels.

The concentration of a scaffold protein is important for titration of signaling partner proteins since excessive concentrations of the scaffold protein result in attenuation of signaling strength [27]. As the concentration of the scaffold protein increases, it plays as a platform to enhance interaction between signaling partner proteins, which leads to increased signaling activity. However, when the scaffold protein is present in excess of the signaling partner proteins, the scaffold protein will separate signaling partner proteins into different complexes, thus preventing the formation of active complexes (Fig 4A). Therefore, we investigated whether an increase in DUSP22 concentration has a biphasic effect on the interaction between signaling proteins. An increase in DUSP22 expression resulted first in an increase in the interaction between ASK1 and MKK7 increased, and a subsequent decrease (Fig 4B).

This biphasic effect was also evident when we measured JNK phosphorylation levels by means of DUSP22 concentration-dependent titration. We examined the levels of p-JNK in a DUSP22 WT dose-dependent manner by immunoblot analysis using an anti-p-JNK antibody. As shown in Fig 4C, the endogenous p-JNK levels increased with increasing DUSP22 concentration, and then gradually decreased at higher concentrations of DUSP22. Similar results were obtained using cells transfected with the DUSP22 C88S mutant plasmid (Fig 4D). These results imply that DUSP22 plays a scaffold role in JNK signal transduction in a phosphatase activity-independent manner.

### DUSP22 enhances apoptosis independently of its phosphatase activity

**Fig 5:**
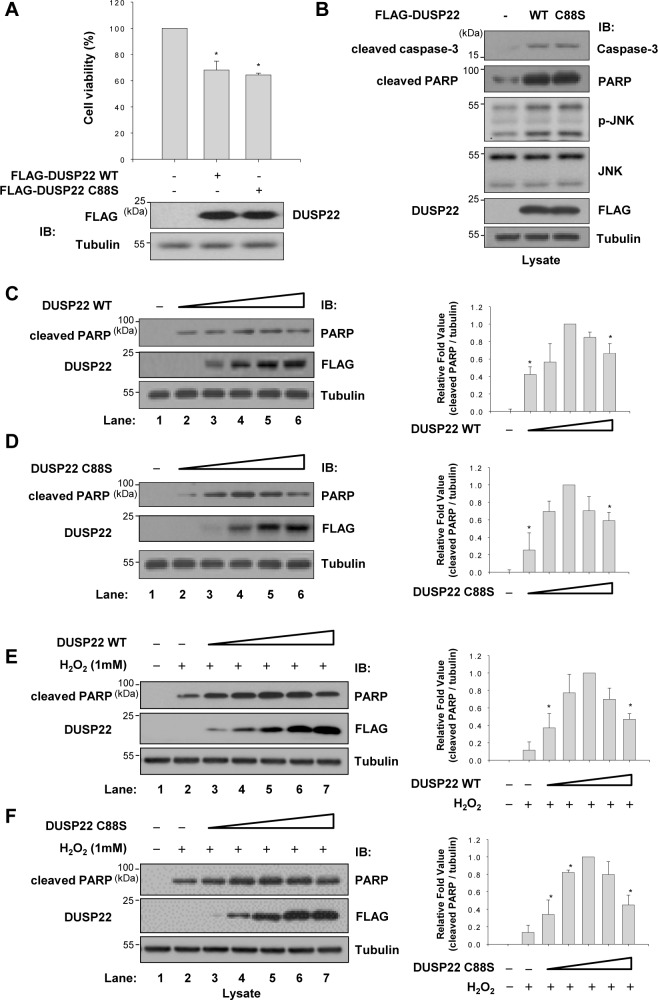
DUSP22-induced cell death. (A) Cell viability was determined as described in Materials and Methods. HEK 293 cells were transfected with FLAG-DUSP22 (WT or C88S mutant, 1 μg) expression plasmid. Data represent means ± SEM of six observations from three independent experiments. The expression levels of DUSP22 were determined by immunoblotting with an anti-FLAG antibody. IB, immunoblot. *p < 0.01 versus the control sample by Student’s *t*-test. (B) HCT 116 cells were transfected with 2 μg of FLAG-DUSP22 WT or FLAG-DUSP22 C88S expression plasmid. After 48 h of transfection, cell lysates were subjected to immunoblotting with indicated antibodies. Fifty microgram of cell lysate was loaded in each lane and the blot was probed with antibodies specific to p-JNK, cleaved PARP, or cleaved caspase-3. Twenty microgram of cell lysate was used for detection of JNK and FLAG. HCT 116 cells were transfected with (C) and (E) FLAG-DUSP22 WT (0, 0.5, 1, 2, 3, 4 μg) or (D) and (F) FLAG-DUSP22 C88S (0, 0.5, 1, 2, 3, 4 μg) for 48 h and then incubated for 1 h in the absence or presence of H_2_O_2_. Total cell lysates were subjected to immunoblot analyses with appropriate antibodies. Right panel: Relative fold of cleaved PARP levels after normalization to corresponding tubulin levels. All data are representative of three independent experiments.

Since DUSP22 is associated with the ASK1-mediated JNK signaling pathway, we investigated whether DUSP22 could induce cell death. We first carried out cell viability assays to assess the effect of DUSP22 on cell viability (Fig 5A). Overexpression of DUSP22 in HEK 293 cells resulted in 35% increase of cell death. The phosphatase activity of DUSP22 was not required for cell death since the phosphatase-inactive mutant DUSP22 C88S yielded similar results to the WT.

Because the ASK1-JNK signaling cascade induces mitochondria-dependent apoptosis [28], we examined induction of JNK phosphorylation and caspase activation. As shown in Fig 5B, cells transfected with DUSP22 WT or mutant plasmids showed significantly increased JNK phosphorylation. Simultaneously, cleaved caspase-3 levels were enhanced in the presence of WT and mutant DUSP22. Activated caspase-3 leads to cleavage of poly (ADP-ribose) polymerase (PARP), which contributes to cell death [29]. We also found that DUSP22 induced an increase in the level of cleaved PARP, irrespective of its phosphatase activity. When the DUSP22 expression, however, further increased, cleaved PARP levels reached maximum and then declined. As shown in Fig 5C and 5D, processing of PARP was increased up to 2 μg of each DUSP22 expression plasmid transfected. However, the cleavage levels of PARP were gradually decreased with increasing concentration of DUSP22 in a phosphatase activity-independent manner. This biphasic effect of DUSP22 on PARP cleavage was also observed in cells exposed to H_2_O_2_ that stimulates JNK signaling (Fig 5E and 5F). These results indicate that DUSP22 plays a critical role as a scaffold that regulates cell death as well as JNK activation.

## Discussion

The function of DUSP22 in the regulation of JNK signaling was unclear, although several reports showed that DUSP22 activates JNK [22, 30]. Three hypotheses for enhancement of JNK activation by DUSP22 have been formulated. The first hypothesis is that DUSP22 dephosphorylates phospho-amino acids at the kinase inhibitory site, which is inhibited upon phosphorylation. The second hypothesis is that DUSP22 dephosphorylates and inactivates inhibitors of JNK signaling. The third hypothesis is that DUSP22 acts as a scaffold protein. In this study, we presented novel findings regarding the scaffolding role of DUSP22. A striking feature of scaffold proteins is their assembly of multiple components of the signaling cascade in sequence [31]. We detected physical interactions of DUSP22 with ASK1, MKK7, and JNK1/2 (Figs 2 and 3). Moreover, the enzymatic activity of DUSP22 was not necessary for JNK activation (Fig 1). Recent reports have shown that DUSP19 and DUSP23, members of the atypical DUSP subgroup, are involved in amplification of specific MAPK activation and considered as putative scaffold proteins. DUSP19 biphasically regulates JNK activation by associating with ASK1 and MKK7 [32, 33]. DUSP23 also enhances JNK and p38 activation in response to various cell stresses by inducing phosphorylation of the upstream kinases MKK4 and MKK6 [34]. Both DUSP19 and DUSP23 regulate MAPK activation irrespective of their phosphatase activities. Taking this into account, we suggest that DUSP22 tethers ASK1-MKK7-JNK1/2 modules and facilitates activation of the JNK signaling pathway irrespective of its phosphatase activity.

The balance between the concentration of a scaffold protein and signaling components is critical for optimal signal transduction. Increased expression of scaffold proteins leads to signal amplification. However, excessive expression of the scaffold protein might result in a decrease of the signal by separating the signaling partner proteins [27, 35]. Several research groups have reported that overexpression of scaffold proteins causes inhibition of signal transduction except in the presence of sufficient levels of signaling partner proteins [12, 36]. For example, the scaffolding proteins, JIP-1 and JIP-2, were first discovered as inhibitors of JNK when they were overexpressed [37]. We also observed a biphasic regulation pattern of JNK phosphorylation depending on the levels of DUSP22 expression (Fig 4C and 4D). Furthermore, the association between ASK1 and MKK7 was regulated by DUSP22 in a dose-dependent manner, which was similar to the biphasic regulation pattern of JNK phosphorylation (Fig 4B). Furthermore, MKK4 and MKK7 are known to play different roles in JNK activation in response to extracellular stimuli. MKK7 is primarily activated in response to cytokines, and MKK4 is primarily activated by extracellular stresses such as osmotic shock and UV radiation [38, 39]. Since MKK7 may have stronger interaction with JNK than MKK4 in the presence of DUSP22, our results suggest that DUSP22 may function as a scaffold protein in the cytokine-regulated MKK7-JNK signaling.

In conclusion, these findings indicate that DUSP22 plays a scaffolding role in JNK activation by bringing together ASK1-MKK7-JNK signaling modules. Further understanding of the functional mechanism of DUSP22 in JNK signaling is necessary, but our data strongly suggest that DUSP22 is a key regulator of the amplitude or duration of JNK signaling.
